# Genetic variants in *XPD* gene and glioma susceptibility in Chinese children: A multicenter case–control study

**DOI:** 10.1002/cai2.6

**Published:** 2022-06-30

**Authors:** Yong‐Ping Chen, Yuxiang Liao, Li Yuan, Xiao‐Kai Huang, Ji‐Chen Ruan, Hui‐Ran Lin, Lei Miao, Zhen‐Jian Zhuo

**Affiliations:** ^1^ Department of Pediatric Surgery, Guangzhou Institute of Pediatrics, Guangdong Provincial Key Laboratory of Research in Structural Birth Defect Disease, Guangzhou Women and Children's Medical Center Guangzhou Medical University Guangzhou Guangdong China; ^2^ Department of Neurosurgery, Xiangya Hospital Central South University Changsha China; ^3^ Department of Pathology, Guangzhou Women and Children's Medical Center Guangzhou Medical University Guangzhou Guangdong China; ^4^ Department of Hematology The Second Affiliated Hospital and Yuying Children's Hospital of Wenzhou Medical University Wenzhou Zhejiang China; ^5^ Faculty of Medicine Macau University of Science and Technology Macau China; ^6^ Laboratory Animal Center, School of Chemical Biology and Biotechnology Peking University Shenzhen Graduate School Shenzhen China

**Keywords:** *XPD*, single polymorphism nucleotide, pediatric glioma, susceptibility

## Abstract

**Background:**

Glioma is one of the central nervous system (CNS) tumors in children, accounting for 80% of malignant brain tumors. Nucleotide excision repair (NER) is a vital pathway during DNA damage repair progression. Xeroderma pigmentosum group D (XPD) or excision repair cross‐complementing group 2 (ERCC2) is a critical factor in the NER pathway, playing an indispensable role in the DNA repair process. Therefore, the genetic variants in XPD may be associated with carcinogenesis induced by defects in DNA repair.

**Methods:**

We are the first to conduct a multi‐center case‐control study to investigate the correlation between XPD gene polymorphisms and pediatric glioma risk. We chose three single nucleotide polymorphisms and genotyped them using the TaqMan assay.

**Results:**

Although there is no significant association of these genetic variations with glioma susceptibility, the stratified analysis revealed that in the subtype of astrocytic tumors, the rs13181 TG/GG genotype enhanced glioma risk than the TT genotype, and carriers with two to three genotypes also elevated the tumor risk than 0‐1 genotypes.

**Conclusion:**

In conclusion, our findings provided an insight into the impact of XPD genetic variants on glioma risk.

## Graphical abstract

The multi‐center case‐control study illuminated the association between *XPD* single nucleotide polymorphisms and glioma in Chinese children, with negative outcoming in single‐locus analysis and combined effect analysis. However, the stratified analysis uncovered either *XPD* rs13181 TG/GG genotypes by comparison with TT genotypes, or carriers with 2‐3 genotypes when compared to 0‐1 genotypes, escalating glioma risk in the subgroup of astrocytic tumors.

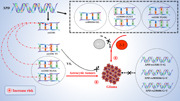

**Abbreviations**
ATRX: alpha‐thalassemia/mental retardation X
CIs: confidence intervals
CNS: central nervous system
ERCC2: excision repair cross‐complementing group 2
GG‐NER: global genome NER
HWE: Hardy–Weinberg equilibrium
MAF: minor allele frequency
MGMT: O^6^‐methylguanine‐DNA methyltransferase
NER: Nucleotide excision repair
NHEJ: nonhomologous end joining
ORs: odds ratios
PHGG: pediatric high‐grade gliomas
PLGG: pediatric low‐grade gliomas
SNPs: single nucleotide  polymorphisms
TC‐NER: transcription‐coupled NER
TERT: telomerase reverse transcriptase
TFIIH: transcription factor IIH
UV‐DDB: UV‐damaged DNA‐binding activity
WHO: World Health Organization
XPD: xeroderma pigmentosum group D

## 1 INTRODUCTION

Glioma is the most frequent tumor in children, originating from glial and precursor cells. Glioma accounts for above 51% of pediatric tumors, and the incidence of malignant glioma has increased for nearly two decades among children aged 0–14 years [1]. Based on the World Health Organization (WHO) classification, gliomas were classified into benign astrocytomas (low‐grade for grades I–II), anaplastic astrocytomas (high‐grade for grade III), and glioblastoma (high‐grade for grade IV) [2]. The most common pediatric gliomas are astrocytomas, oligodendrogliomas, ependymomas, and brainstem gliomas. Among gliomas, pilocytic astrocytoma is considered nonmalignant, recognized by the WHO and clinical practice. Glioblastoma and other diffuse gliomas were categorized into malignancies, frequently occurring in adults [3]. Most gliomas in children were pediatric low‐grade gliomas (PLGG), which developed slowly, referred to as grades I–II by WHO. Meanwhile, some pediatric gliomas, namely high‐grade gliomas (PHGG), were in rapid occurrence and development classified as grades III–IV [4]. The current standard of care for glioma is surgery, radiation, and alkylating agents [5]. However, the overall survival rate of glioma is unsatisfactory. Pediatric gliomas may develop into adult gliomas because of low‐grade lesions and remaining recurrence for many years [6].

DNA repair pathways mainly have nonhomologous end joining (NHEJ), base excision repair (BER), and nucleotide excision repair (NER) [7]. As one of the classical general impaired repair pathways, the NER pathway is highly conservative. Its function is the removal of bulky adducts caused by UV radiation and several chemical agents [8]. In addition, several essential NER genes associated closely with xeroderma pigmentosum (XP) are *XPA*, *XPB*, *XPC*, *XPD*, *XPE*, *XPF*, and *XPG*, respectively. They together ensure the DNA repair procedure [9]. XP group D (XPD/ERCC2), an 87 kDa protein, is a transcription factor IIH (TFIIH) component. *XPD* gene is located at chromosome 19q13.3 and contains 22 exons and 21 introns, encoding 2.3 kb messenger RNA (mRNA) [10].* XPD* played an essential role in the NER pathway because it could encode a core ATP‐dependent DNA helicase and utilize 5′–3′ polarity, unwinding the DNA duplex near impaired sites. However, this XPD helicase is optional for transcription [11]. Additionally, *XPD* was found in mitochondria, and it could keep the mitochondrial genome from being impaired by oxidative DNA [12]. Mutations in *XPD* principally were from single residue alterations, sometimes in neighboring residues [11]. Not only were the mutants in the *XPD* gene associated with XP, trichothiodystrophy, and Cockayne's syndrome, but they affected the cancer susceptibility [13].

It is still unsure whether the numerous genetic variations in many genes are considered the cause or the effect of cancer. If the consideration is the reason, the genetic variants studies benefit enormously from the effect of single nucleotide polymorphisms (SNPs) on cancer occurrence and progression. Growing research demonstrated that genetic modifications in DNA repair genes like SNPs could influence protein expression during repair, containing encoding, transcription, and translation. Finally, these influences impaired DNA repair capacity and led to genetic instability, even carcinogenesis [14]. Given the importance of the *XPD* gene in impacting DNA repair capacity, genetic variants in this core gene were likely to alter cancer risk. For instance, *XPD* rs1799787 is closely associated with lung cancer risk, and the combined effect with other NER genes and SNPs contributed to elevated lung cancer risk [15]. Variant alleles in *XPD* also enhanced pancreatic cancer susceptibility [16]. Recent genome‐wide association studies have identified 13 new loci for glioma, such as polymorphisms in RAVER2 (rs12752552), MDM4 (rs4252707), and AKT3 (rs12076373) [17]. However, the relationship between *XPD* gene polymorphisms and pediatric glioma risk has not been studied. Therefore, we conducted a case–control study to explore the impact of *XPD* SNPs on glioma susceptibility in Chinese children for the first time.

## 2 MATERIALS AND METHODS

### 2.1 Study subjects

This case‐control study included 314 glioma cases and 380 healthy controls, which were collected from Guangzhou Women and Children's Medical Center (171 cases and 228 controls), Xiangya Hospital (85 cases and 132 controls), and the Second Affiliated Hospital and Yuying Children's Hospital of Wenzhou Medical University (58 cases and 20 controls). The cases were confirmed as patients with glioma. The controls group was healthy participants recruited randomly from hospital visitors during the same period as the cases. They were matched based on age and gender distribution. All the participants signed informed consent to use their samples before the study. Our research was approved by the institutional review board of Guangzhou Women and Children's Medical Center.

We selected potentially functional SNPs from NCBI dbSNP database (https://www.ncbi.nlm.nih.gov/snp/) and SNPinfo (https://snpinfo.niehs.nih.gov). In brief, the selection criteria for candidate SNPs should be met: the minor allele frequency (MAF) in the Chinese Han population should be more than 5%; SNPs were limited to 3′UTR, 5′UTR, coding region, and upstream promoter region of *XPD* gene; the chosen SNPs showed low linkage disequilibrium (*R* ^2^ < 0.8). Ultimately, three *XPD* SNPs (rs3810366, rs238406, rs13181) were selected. The rs13181 is likely to affect the exonic splicing enhancer or exonic splicing silencer, and splicing abolishes domain, and nonsynonymous coding SNP (nsSNP). The rs238406 also has the potential function to influence exonic splicing enhancer or exonic splicing silencer. Additionally, rs3810366 may influence the activity of transcription factor binding sites. DNA was extracted from blood samples with the QIAamp DNA Blood Mini kit (QIAGEN). Selected SNPs were genotyped using TaqMan real‐time PCR (Applied Biosystems). The details for genotyping are the following conditions: preread stage at 60℃ for 30 s, holding stage at 95℃ 10 min, repeated 45 cycles each of denaturation at 95℃ for 15 s, annealing, and extension at 60℃ for 1 min. Then, we selected the standard run mode and added the reaction volume (5 µl for each well in a 384‐well reaction plate) into the instrument. Finally, we loaded the reaction plate, then started the run, genotyping 10% of the samples randomly and blindly, exhibiting a 100% concordance rate.

### 2.2 Statistical analysis

Using the goodness‐of‐kit *χ* ^2^ test among control subjects to determine whether genotypes complied with Hardy–Weinberg equilibrium (HWE), we employed the *χ* ^2^ test or *t*‐test to compare the differences in clinical variables between the cases and controls. Multivariate logistic regression analysis was performed to estimate the association between the SNPs and glioma risk by the age‐ and gender‐adjusted odds ratios (ORs) and 95% confidence intervals (CIs). We further conducted the stratification analysis for cases in age, gender, subtypes, and clinical‐stage subgroups. All the statistical analyses were completed using the SAS v10.0 (SAS Institute Inc.), and a two‐sided *p* < 0.05 was considered significant. To compare the XPD expression between glioma groups from WHO grades II‐IV, we utilized one‐way ANOVA by online analysis from the Chinese Glioma Genome Atlas (CGGA).

## 3 RESULTS

### 3.1 Characteristics of study participants

Detailed population characteristics between 314 cases and 380 controls are shown in Supporting Information: Table S1. Cases and controls were well matched by age (*p* = 0.461) and gender (*p* = 0.379), with no statistically significant difference. Among the patients, the astrocytic tumors (214) accounted for 68.15%, the ependymoma (61) for 19.43%, the neuronal and mixed neuronal‐glial tumors (25) for 7.96%, the embryonal tumors (12) for 3.82%, and 2 for 0.64% could not be classified. Cases were staged by WHO classification, including 151 (48.09%) for grade I, 73 (23.23%) for grade II, 36 (11.46%) for grade III, and 53 (16.88%) for grade IV.

Three SNPs in the *XPD* gene were genotyped successfully in 313 cases and 380 controls, as presented in Table 1. Regrettably, we could not find significant associations between selected *XPD* gene polymorphisms and glioma risk in single‐locus and combined effect analyses.

**Table 1:** Association of *XPD* gene polymorphisms with glioma susceptibility in Chinese children

Genotype	Cases (*N* = 313)	Controls (*N* = 380)	*p* a	Crude OR (95% CI)	*p*	Adjusted OR (95% CI)^b^	*p* b
rs3810366 G > C (HWE = 0.603)							
GG	86 (27.48)	96 (25.26)		1.00		1.00	
GC	156 (49.84)	195 (51.32)		0.89 (0.62–1.28)	0.536	0.88 (0.61–1.26)	0.484
CC	71 (22.68)	89 (23.42)		0.89 (0.58–1.36)	0.593	0.89 (0.58–1.36)	0.581
Additive			0.582	0.94 (0.76–1.17)	0.582	0.94 (0.76–1.16)	0.566
Dominant	227 (72.52)	284 (74.74)	0.510	0.89 (0.64–1.25)	0.509	0.88 (0.63–1.24)	0.468
GG/GC	242 (77.32)	291 (76.58)		1.00		1.00	
CC	71 (22.68)	89 (23.42)	0.819	0.96 (0.67–1.37)	0.819	0.97 (0.68–1.38)	0.844
rs238406 G > T (HWE = 0.728)							
GG	88 (28.12)	102 (26.84)		1.00		1.00	
GT	160 (51.12)	193 (50.79)		0.96 (0.67–1.37)	0.825	0.95 (0.67–1.35)	0.769
TT	65 (20.77)	85 (22.37)		0.89 (0.58–1.36)	0.584	0.88 (0.57–1.36)	0.564
Additive			0.590	0.94 (0.76–1.17)	0.590	0.94 (0.76–1.17)	0.567
Dominant	225 (71.88)	278 (73.16)	0.709	0.94 (0.67–1.31)	0.708	0.93 (0.66–1.30)	0.661
GG/GT	248 (79.23)	295 (77.63)		1.00		1.00	
TT	65 (20.77)	85 (22.37)	0.610	0.91 (0.63‐1.31)	0.612	0.91 (0.63‐1.31)	0.620
rs13181 T > G (HWE = 0.813)							
TT	261 (83.39)	331 (87.11)		1.00		1.00	
TG	50 (15.97)	47 (12.37)		1.35 (0.88–2.07)	0.172	1.34 (0.87–2.06)	0.190
GG	2 (0.64)	2 (0.53)		1.27 (0.18–9.06)	0.813	1.33 (0.19–9.56)	0.777
Additive			0.180	1.31 (0.88–1.96)	0.181	1.31 (0.88–1.95)	0.192
Dominant	52 (16.61)	49 (12.89)	0.167	1.35 (0.88–2.05)	0.168	1.34 (0.87–2.04)	0.182
TT/TG	311 (99.36)	378 (99.47)		1.00		1.00	
GG	2 (0.64)	2 (0.53)	0.846	1.22 (0.17–8.69)	0.845	1.28 (0.18–9.18)	0.807
Combined effect of risk genotypes^c^							
0	3 (0.96)	1 (0.26)		1.00		1.00	
1	249 (79.55)	325 (85.53)		0.26 (0.03–2.47)	0.239	0.30 (0.03–2.90)	0.296
2	46 (14.70)	47 (12.37)		0.33 (0.03–3.26)	0.340	0.37 (0.04–3.76)	0.404
3	15 (4.79)	7 (1.84)	0.040	0.72 (0.06–8.16)	0.787	0.85 (0.07–9.77)	0.894
0–1	252 (80.51)	326 (85.79)		1.00		1.00	
2–3	61 (19.49)	54 (14.21)	0.063	1.46 (0.98–2.18)	0.064	1.46 (0.97–2.18)	0.069

Abbreviations: CC, homozygous cytosine; CI, confidence interval; GC, heterozygote of guanine and cytosine; GG, homozygous guanine; GT, heterozygote of guanine and thymine; HWE, Hardy–Weinberg equilibrium; OR, odds ratio; TG, heterozygote of thymine and guanine; TT, homozygous thymine.

^a^ *χ* ^2^ test for genotype distributions between glioma patients and cancer‐free controls.

^b^ Adjusted for age and sex.

^c^ Risk genotypes were carriers with rs3810366 GG, rs238406 GG/GT, and rs13181 TG/GG genotypes.

### 3.2 Stratification analysis

We conducted the stratified analysis by age, gender, subtypes, and clinical‐stage subgroups. The *XPD* rs13181 heterozygote of thymine and guanine/homozygous guanine (TG/GG) genotypes was observed with enhanced glioma risk in astrocytic tumors when compared to homozygous thymine genotypes (adjusted OR = 1.60, 95% CI = 1.02–2.54, *p* = 0.043). Furthermore, rs3810366 GG, rs238406 homozygous guanine/heterozygote of guanine and thymine (GG/GT), and rs13181 TG/GG genotypes were referred to as risk genotypes. In comparison to 0–1 genotypes, carriers with two to three genotypes were associated with increased glioma risk in the subtype of astrocytic tumors (adjusted OR = 1.69, 95% CI = 1.09–2.62, *p* = 0.019) (Table 2).

**Table 2:** Stratification analysis of risk genotypes with glioma susceptibility

	rs13181 (cases/controls)				Risk genotypes (cases/controls)			
Variables	TT	TG/GG	AOR (95% CI)^a^	*p* a	0–1	2–3	AOR (95% CI)^a^	*p* a
Age, month								
<60	113/150	21/24	1.16 (0.62–2.19)	0.641	109/147	25/27	1.25 (0.69–2.28)	0.459
≥60	148/181	31/25	1.54 (0.87–2.72)	0.142	143/179	36/27	1.69 (0.98–2.92)	0.060
Sex								
Females	123/145	23/19	1.39 (0.72–2.68)	0.328	118/145	28/19	1.76 (0.93–3.32)	0.080
Males	138/186	29/30	1.30 (0.74–2.26)	0.362	134/181	33/35	1.27 (0.75–2.15)	0.373
Subtypes								
Astrocytic tumors	172/331	42/49	**1.60 (1.02**–**2.54)**	**0.043**	166/326	48/54	**1.69 (1.09**–**2.62)**	**0.019**
Ependymoma	53/331	7/49	0.92 (0.39–2.16)	0.841	50/326	10/54	1.21 (0.57–2.56)	0.627
Neuronal and mixed	24/331	1/49	0.28 (0.04–2.10)	0.214	24/326	1/54	0.24 (0.03–1.84)	0.170
Embryonal tumors	10/331	2/49	1.50 (0.31–7.26)	0.613	10/326	2/54	1.40 (0.29–6.77)	0.675
Clinical stage								
I	123/331	28/49	1.52 (0.91–2.55)	0.109	120/326	31/54	1.55 (0.95–2.55)	0.081
II	65/331	7/49	0.72 (0.31–1.67)	0.449	60/326	12/54	1.20 (0.61–2.38)	0.600
III	31/331	5/49	1.09 (0.40–2.98)	0.862	30/326	6/54	1.18 (0.46–3.02)	0.724
IV	41/331	12/49	2.01 (0.97–4.18)	0.061	41/326	12/54	1.83 (0.89–3.78)	0.102
I + II	188/331	35/49	1.25 (0.78–2.00)	0.362	180/326	43/54	1.43 (0.92–2.23)	0.114
III + IV	72/331	17/49	1.59 (0.87–2.93)	0.134	71/326	18/54	1.55 (0.86–2.81)	0.149

*Note*: The statistically significant values are bold with *p* < 0.05.

Abbreviations: CI, confidence interval; GG, homozygous guanine; OR, odds ratio; TG, heterozygote of thymine and guanine; TT, homozygous thymine.

^a^ Adjusted for age and sex, omitting the corresponding stratify factor.

**Figure 1:**
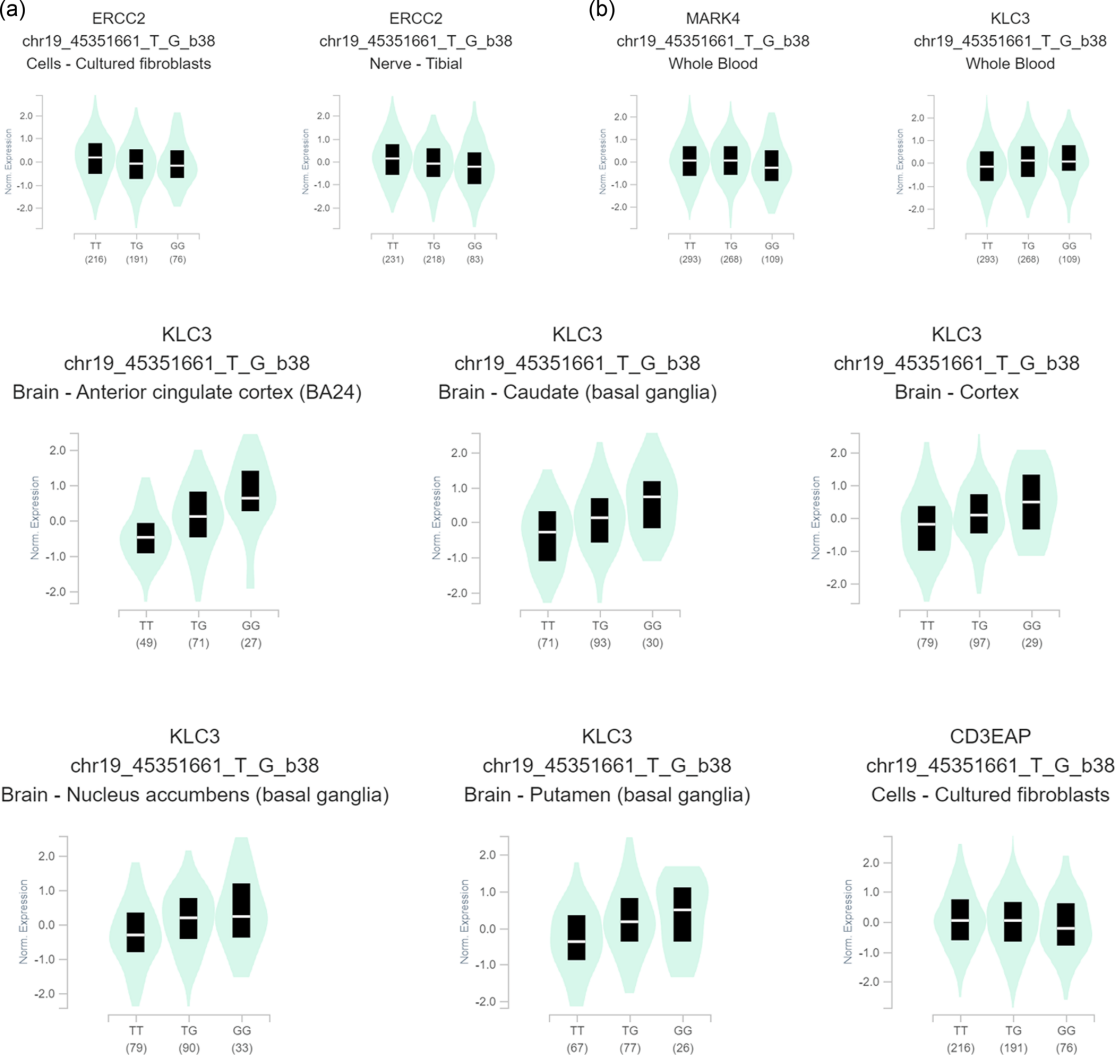
Functional effect of *XPD *gene rs13181 polymorphism from GTEx portal. (a) Demonstrates the messenger RNA (mRNA) expression of *XPD* rs13181 genotypes in cell‐cultured fibroblasts and nerve‐tibial. (b) Explicates different mRNA expressions of its vicinal genes *KLC3*, *CD3EAP*, and *MARK4* in different tissues.

We gained the published functional relevance from GTEx (http://www.gtexportal.org/home/) and evaluated the mRNA levels varied with *XPD* genotypes. As denoted in Figure 1a, the rs13181 G genotype dipped *XPD* mRNA levels conspicuously in cell‐cultured fibroblasts and nerve‐tibial than the rs13181 T genotype. Simultaneously, the rs13181 G genotype altered mRNA levels of genes in the vicinity, involving *KLC3*, *CD3EAP*, and *MARK4*.

### 3.3 Functional annotation of *XPD*

**Figure 2:**
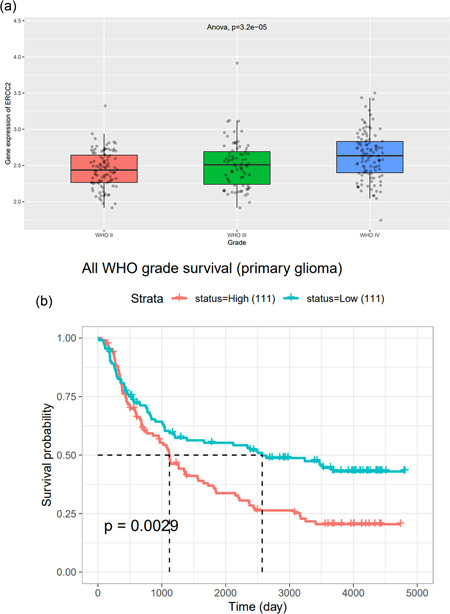
The association between *XPD *expression and glioma development from the Chinese Glioma Genome Atlas database. (a) Manifests the relevance of *XPD* gene expression on glioma (classified by World Health Organization grade). (b) Kaplan‐Meier method was used for survival analysis, revealing the distinguished patient survival by *XPD* gene expression.

We further acquired outright data for functional annotation of *XPD* in the mRNAseq_325 data set (www.cgga.org.cn) from the Chinese Glioma Genome Atlas (CGGA). Through one‐way ANOVA, the outcome illuminated that these was a statistical difference in the XPD gene expression between WHO grades II‐IV (Figure 2a). The survival analysis was also implemented to scrutinize the correlation between *XPD* expression and survival probability, which indicated that strengthened *XPD* expression threatened glioma patients (Figure 2b). These results might conduce to discover the *XPD* implication in pediatric glioma.

## 4 DISCUSSION

The existing problems, such as complicated treatment and poor prognosis in glioma, have disturbed children's health. Glioma has discrepancies between children and adults at the molecular level. Additionally, different from adult glioma, most pediatric glioma belongs to WHO grade I. Thus, although the molecular pathogenesis research in adult glioma has grown tremendously, these genetic findings may not explain the etiology of pediatric glioma. In addition, how the *XPD* polymorphisms impact the underlying molecular mechanism of glioma tumorigenesis in children still has not been entirely investigated. To systematically discover the strength of associations between *XPD* gene SNPs and glioma risk in Chinese children for the first time, we performed a multicenter case–control study with 314 cases and 380 controls. Moreover, we also evaluated the glioma risk in carriers with SNPs risk genotypes.

Various environmental factors have been reported to influence glioma, among which the effect of ionizing radiation was confirmed [18]. Genetic factors also occupied an essential role, and many molecular diagnostic markers in glioma have been discovered, such as *IDH*, codeletion of chromosomal arms 1p and 19q (1p/19q codeletion), *H3F3A*, nuclear alpha‐thalassemia/mental retardation X‐linked syndrome (*ATRX*) gene, O^6^‐methylguanine‐DNA methyltransferase (*MGMT*), and telomerase reverse transcriptase (*TERT*) [19]. Many DNA repair mechanisms are categorized as direct and indirect repair types [20]. The NER pathway belongs to one of the excision repair systems in the indirect repair mechanisms, which completes repair progress by excising and removing about 24–32nt impaired DNA fragments after DNA replication. The most frequent DNA damage, processed by NER, is bulky adduct from DNA mutations due to mutagenic agent exposure [21]. The dysfunctional NER pathway would influence cancer risk. In a study corresponding to colorectal cancer in the Chinese population, some NER genetic variants (*XPA* rs10817938 and *XPC* rs2607775) were illustrated to alter disease risk. Moreover, this study revealed that genetic biomarkers in the NER pathway were associated with predicting colorectal cancer risk and clinical outcome [22]. Recent research elaborated that the disequilibrium of the NER pathway increased the risk of hematological malignancies among patients with XP group C. In these patients, bulky purine DNA lesions could not be repaired entirely, leading to multiple mutations in transcription and replication, consequently the higher risk of internal cancers [23]. There are two subpathways in the NER pathway: global genome NER (GG‐NER) and transcription‐coupled NER (TC‐NER). The GG‐NER is used for whole‐genome repair. First, it mainly relies on the XPC‐RAD23B complex and UV‐DDB (UV‐damaged DNA‐binding activity) to complete damage identification. Then, it unwinds and stabilizes impaired DNA through XPG, XPB, and XPD. Subsequently, it cleaved the damaged DNA fragment mainly with the help of XPG, ERCC1‐XPF, and finally used various DNA polymerases to fill the gaps [24]. Besides, when its defects, it can result in cancer predisposition. The TC‐NER is initiated by the suppressed RNA polymerase, which is indispensable for damage recognition, occurring at the damaged transcriptional strand in active genes. It works through combination with transcription mechanisms, playing a role in numerous syndromes [8, 25].

DNA repair pathway maintains genome stability by protecting the genome from damage by endogenous and exogenous hazardous agents, such as environmental and genetic factors and their interactions. The variants in DNA repair genes may alter cancer risk [7]. Especially, *XPD* mediated various biological functions to stimulate disease growth and development [26]. There are many SNPs in the *XPD* gene impacting genic exons and introns. Some nonsynonymous SNPs may affect gene encoding ability and protein expression levels [27]. Some *XPD* polymorphisms have been suggested to modulate DNA repair capacity and promote tumorigenesis [28].* XPD* Asp312Asn (rs1799793) was related to prostate cancer pathology [29].* XPD* Lys751Gln (rs13181) polymorphism could increase the susceptibility of such cancers [30]. Of note, genetic variations that arise spontaneously, referred to as SNPs, showed potential for predicting the glioma risk [18]. Moreover, several *XPD* gene polymorphisms may influence the DNA repair ability in glioma [27]. Our chosen SNPs are rs3810366, rs238406, and rs13181, all in chromosome 19. The rs3810366 polymorphism is at the position of 45370684 with the G/A/C/T substitution. The rs238406 alleles variants T/G are at 45365051 in exon 6. The rs13181 T/A/G is located in 45351661 in exon 23, contributing to an amino acid transformation from Lys to Gln. Of them, *XPD* rs13181 is the most common variant. In addition, the rs13181 plays a prominent role in the work of XPD helicase via influencing the C‐terminal domain [31]. These SNPs may modify cancer risk and be treated as potential markers of carcinogenesis [32]. In a correlation study from Poland, the DNA repair gene polymorphisms have significant associations with breast cancer risk in Polish women. Specifically, the *XPD* rs13181 allele strongly promoted the risk of breast cancer [33]. However, our previous study could not find evidence that *XPD* genetic variations are associated with hepatoblastoma susceptibility in a single locus analysis [34]. Worthy of note, *XPD* rs3810366 and rs238406 were identified to alter neuroblastoma risk. *XPD* rs3810366 could improve the susceptibility of neuroblastoma, while the protective effect of *XPD* rs238406 on neuroblastoma tumorigenesis was detected [35]. Furthermore, Zhang et al. revealed that the essential prognostic factors for the lung cancer risk might be *XPD* rs13181 T > G and rs1799793 C > T. They found that both SNPs could enhance death risk, and the role of *XPD* rs13181 was more prominent in age, sex, and smoking [36]. Maral Adel Fahmideh et al. conducted a systematic review and meta‐analysis. They observed that the *XPD* rs13181 might be more susceptible to glioma risk, but no evidence of a significant relationship with glioma susceptibility has been accessed for another polymorphism of *XPD*, rs1799793 [37]. However, no significant SNPs with glioma susceptibility were identified among the results in any statistical analysis but stratified analysis in our study. The stratified analysis demonstrated *XPD* rs13181 TG/GG and individuals with two to three risk genotypes promoted disease risk in astrocytic tumors. The interpretations of negative results may include inadequate sample size, population heterogeneity, and low‐penetrance of a single variant. In a previous study, DNA repair gene *XPD* was considered a low‐penetrance gene for glioma susceptibility [37]. Existing research has elucidated the effect of *XPD* rs13181 on the risk of glioma [37], but has not yet probed into the correlation of *XPD* rs3810366 with glioma risk, so our negative result regarding rs3810366 will provide an insight into the potential role of this SNP in glioma etiology. Another negative outcome of our research was that the impact of rs238406 on glioma risk was unsuccessfully assessed in one comprehensive analysis and two meta‐analyses [38, 39, 40].

Several limitations can be noted. First, an individual's cancer susceptibility may be an accumulative effect of various risk factors. Our research only concentrated on genes, without considerations like environmental agents or gene–environment interactions. Second, we only selected three SNPs in the *XPD* gene for the association research, and more potentially functional polymorphisms should be taken into further exploration. Third, a limited sample size might weaken the statistical power, and thereby the strength of correlation could not be objectively reflected. Fourth, the subjects were recruited from the Chinese Han children, so the ethnic discrepancy should be accounted for by applying results to other ethnicities. Finally, we did not conduct relevant biological experiments to validate the related protein and RNA expression levels, which should be further studied.

In sum, this study investigated the potential effect of *XPD* polymorphisms on the glioma risk in children. Our present evidence failed to observe any association between single SNP and glioma risk. However, we detect that the *XPD* rs13181 TG/GG and relevant variant genotypes might elevate glioma susceptibility in a specific subgroup. Larger scale studies are warranted to illuminate the underlying molecular mechanism of how *XPD* genetic variants affect glioma risk.

## AUTHOR CONTRIBUTIONS

**Yong‐Ping Chen**: Investigation (equal); writing—original draft (equal). **Yuxiang Liao**: Data curation (equal). **Li Yuan**: Methodology (equal); writing—review and editing (equal). **Xiao‐Kai Huang**: Data curation (equal); software (equal). **Ji‐Chen Ruan**: Validation (equal). **Hui‐Ran Lin**: methodology (equal); writing—review and editing (equal). **Lei Miao**: Conceptualization (equal); visualization (equal); writing—review and editing (equal). **Zhen‐Jian Zhuo**: Conceptualization (equal); project administration (equal); writing—review and editing (equal).

## CONFLICT OF INTEREST

All authors declare that there is no conflict of interest except Professor Zhenjian Zhuo, who is member of Cancer Innovation Editorial Board. To minimize bias, he was excluded from all editorial decision‐making related to the acceptance of this article for publication.

## ETHICS STATEMENT

This study was approved by Ethics Committee of Guangzhou Women and Children's Medical Center (2016021650).

## INFORMED CONSENT

Not applicable.

## Supporting information

Supporting information.

## Data Availability

The data set analyzed during the current study is available in the repositories: GTEx (http://www.gtexportal.org/home/), mRNAseq_325 data set (www.cgga.org.cn), NCBI dbSNP database (https://www.ncbi.nlm.nih.gov/snp/), and SNPinfo (https://snpinfo.niehs.nih.gov).

## References

[1] Ostrom QT , Cioffi G , Waite K , Kruchko C , Barnholtz‐Sloan JS . CBTRUS statistical report: primary brain and other central nervous system tumors diagnosed in the United States in 2014‐2018. Neuro Oncol. 2021;23(Suppl 3):iii1–105. 10.1093/neuonc/noab200 34608945 PMC8491279

[2] Louis DN , Perry A , Reifenberger G , von Deimling A , Figarella‐Branger D , Cavenee WK , et al. The 2016 World Health Organization Classification of Tumors of the Central Nervous System: a summary. Acta Neuropathol. 2016;131(6):803–20. 10.1007/s00401-016-1545-1 27157931

[3] Miller KD , Ostrom QT , Kruchko C , Patil N , Tihan T , Cioffi G , et al. Brain and other central nervous system tumor statistics, 2021. CA Cancer J Clin. 2021;71(5):381–406. 10.3322/caac.21693 34427324

[4] Sturm D , Pfister SM , Jones DTW . Pediatric gliomas: current concepts on diagnosis, biology, and clinical management. J Clin Oncol. 2017;35(21):2370–7. 10.1200/jco.2017.73.0242 28640698

[5] Touat M , Li YY , Boynton AN , Spurr LF , Iorgulescu JB , Bohrson CL , et al. Mechanisms and therapeutic implications of hypermutation in gliomas. Nature. 2020;580(7804):517–23. 10.1038/s41586-020-2209-9 32322066 PMC8235024

[6] Armstrong GT , Liu Q , Yasui Y , Huang S , Ness KK , Leisenring W , et al. Long‐term outcomes among adult survivors of childhood central nervous system malignancies in the childhood cancer survivor study. J Natl Cancer Inst. 2009;101(13):946–58. 10.1093/jnci/djp148 19535780 PMC2704230

[7] Knijnenburg TA , Wang L , Zimmermann MT , Chambwe N , Gao GF , Cherniack AD , et al. Genomic and molecular landscape of DNA damage repair deficiency across the cancer genome atlas. Cell Rep. 2018;23(1):239–54.e6. 10.1016/j.celrep.2018.03.076 29617664 PMC5961503

[8] Marteijn JA , Lans H , Vermeulen W , Hoeijmakers JH . Understanding nucleotide excision repair and its roles in cancer and ageing. Nat Rev Mol Cell Biol. 2014;15(7):465–81. 10.1038/nrm3822 24954209

[9] Cleaver JE . Common pathways for ultraviolet skin carcinogenesis in the repair and replication defective groups of xeroderma pigmentosum. J Dermatol Sci. 2000;23(1):1–11. 10.1016/s0923-1811(99)00088-2 10699759

[10] Sung P , Bailly V , Weber C , Thompson LH , Prakash L , Prakash S . Human xeroderma pigmentosum group D gene encodes a DNA helicase. Nature. 1993;365(6449):852–5. 10.1038/365852a0 8413672

[11] Fan L , Fuss JO , Cheng QJ , Arvai AS , Hammel M , Roberts VA , et al. XPD helicase structures and activities: insights into the cancer and aging phenotypes from XPD mutations. Cell. 2008;133(5):789–800. 10.1016/j.cell.2008.04.030 18510924 PMC3055247

[12] Liu J , Fang H , Chi Z , Wu Z , Wei D , Mo D , et al. XPD localizes in mitochondria and protects the mitochondrial genome from oxidative DNA damage. Nucleic Acids Res. 2015;43(11):5476–88. 10.1093/nar/gkv472 25969448 PMC4477675

[13] Liu H , Rudolf J , Johnson KA , McMahon SA , Oke M , Carter L , et al. Structure of the DNA repair helicase XPD. Cell. 2008;133(5):801–12. 10.1016/j.cell.2008.04.029 18510925 PMC3326533

[14] He J , Shi TY , Zhu ML , Wang MY , Li QX , Wei QY . Associations of Lys939Gln and Ala499Val polymorphisms of the XPC gene with cancer susceptibility: a meta‐analysis. Int J Cancer. 2013;133(8):1765–75. 10.1002/ijc.28089 23400628

[15] Mei C , Hou M , Guo S , Hua F , Zheng D , Xu F , et al. Polymorphisms in DNA repair genes of XRCC1, XPA, XPC, XPD and associations with lung cancer risk in Chinese people. Thorac Cancer. 2014;5(3):232–42. 10.1111/1759-7714.12073 26767006 PMC4704308

[16] McWilliams RR , Bamlet WR , Cunningham JM , Goode EL , de Andrade M , Boardman LA , et al. Polymorphisms in DNA repair genes, smoking, and pancreatic adenocarcinoma risk. Cancer Res. 2008;68(12):4928–35. 10.1158/0008-5472.Can-07-5539 18544627 PMC2652067

[17] Melin BS , Barnholtz‐Sloan JS , Wrensch MR , Johansen C , Il'yasova D , Kinnersley B , et al. Genome‐wide association study of glioma subtypes identifies specific differences in genetic susceptibility to glioblastoma and non‐glioblastoma tumors. Nat Genet. 2017;49(5):789–94. 10.1038/ng.3823 28346443 PMC5558246

[18] Savage N . Searching for the roots of brain cancer. Nature. 2018;561(7724):S50–1. 10.1038/d41586-018-06709-2 30258163

[19] Louis DN , Perry A , Wesseling P , Brat DJ , Cree IA , Figarella‐Branger D , et al. The 2021 WHO classification of tumors of the central nervous system: a summary. Neuro Oncol. 2021;23(8):1231–51. 10.1093/neuonc/noab106 34185076 PMC8328013

[20] Gatzidou E , Michailidi C , Tseleni‐Balafouta S , Theocharis S . An epitome of DNA repair related genes and mechanisms in thyroid carcinoma. Cancer Lett. 2010;290(2):139–47. 10.1016/j.canlet.2009.08.007 19709803

[21] Gillet LC , Schärer OD . Molecular mechanisms of mammalian global genome nucleotide excision repair. Chem Rev. 2006;106(2):253–76. 10.1021/cr040483f 16464005

[22] Li YK , Xu Q , Sun LP , Gong YH , Jing JJ , Xing CZ , et al. Nucleotide excision repair pathway gene polymorphisms are associated with risk and prognosis of colorectal cancer. World J Gastroenterol. 2020;26(3):307–23. 10.3748/wjg.v26.i3.307 31988591 PMC6969885

[23] Yurchenko AA , Padioleau I , Matkarimov BT , Soulier J , Sarasin A , Nikolaev S . XPC deficiency increases risk of hematologic malignancies through mutator phenotype and characteristic mutational signature. Nat Commun. 2020;11(1):5834. 10.1038/s41467-020-19633-9 33203900 PMC7672101

[24] Kamileri I , Karakasilioti I , Garinis GA . Nucleotide excision repair: new tricks with old bricks. Trends Genet. 2012;28(11):566–73. 10.1016/j.tig.2012.06.004 22824526

[25] Marini F , Nardo T , Giannattasio M , Minuzzo M , Stefanini M , Plevani P , et al. DNA nucleotide excision repair‐dependent signaling to checkpoint activation. Proc Natl Acad Sci USA. 2006;103(46):17325–30. 10.1073/pnas.0605446103 17088560 PMC1859929

[26] Houten BV , Kuper J , Kisker C . Role of XPD in cellular functions: to TFIIH and beyond. DNA Repair (Amst). 2016;44:136–42. 10.1016/j.dnarep.2016.05.019 27262611

[27] Chen H , Shao C , Shi H , Mu Y , Sai K , Chen Z . Single nucleotide polymorphisms and expression of ERCC1 and ERCC2 vis‐à‐vis chemotherapy drug cytotoxicity in human glioma. J Neurooncol. 2007;82(3):257–62. 10.1007/s11060-006-9290-2 17151930

[28] Spitz MR , Wu X , Wang Y , Wang LE , Shete S , Amos CI , et al. Modulation of nucleotide excision repair capacity by XPD polymorphisms in lung cancer patients. Cancer Res. 2001;61(4):1354–7. 11245433

[29] Liao SG , Liu L , Wang Y , Zhang YY , Wang YJ . XPD Asp312Asn polymorphism is a risk factor for prostate cancer. J Cancer Res Clin Oncol. 2012;138(10):1689–95. 10.1007/s00432-012-1246-7 22644997 PMC11824463

[30] Bănescu C , Trifa AP , Demian S , Benedek Lazar E , Dima D , Duicu C , et al. Polymorphism of XRCC1, XRCC3, and XPD genes and risk of chronic myeloid leukemia. BioMed Res Int. 2014;2014:213790. 10.1155/2014/213790 24955348 PMC4052066

[31] Coin F , Bergmann E , Tremeau‐Bravard A , Egly JM . Mutations in XPB and XPD helicases found in xeroderma pigmentosum patients impair the transcription function of TFIIH. EMBO J. 1999;18(5):1357–66. 10.1093/emboj/18.5.1357 10064601 PMC1171225

[32] Wu H , Li S , Hu X , Qin W , Wang Y , Sun T , et al. Associations of mRNA expression of DNA repair genes and genetic polymorphisms with cancer risk: a bioinformatics analysis and meta‐analysis. J Cancer. 2019;10(16):3593–607. 10.7150/jca.30975 31333776 PMC6636297

[33] Smolarz B , Bryś M , Forma E , Zadrożny M , Bieńkiewicz J , Romanowicz H . Data on single nucleotide polymorphism of DNA repair genes and breast cancer risk from Poland. Pathol Oncol Res. 2019;25(4):1311–7. 10.1007/s12253-017-0370-8 29209986

[34] Zhuo Z , Miao L , Hua W , Chen H , Yang Z , Li Y , et al. Genetic variations in nucleotide excision repair pathway genes and hepatoblastoma susceptibility. Int J Cancer. 2021;149(9):1649–58. 10.1002/ijc.33722 34196959

[35] Zhou C , Wang Y , He L , Zhu J , Li J , Tang Y , et al. Association between NER pathway gene polymorphisms and neuroblastoma risk in an eastern Chinese population. Mol Ther Oncolytics. 2021;20:3–11. 10.1016/j.omto.2020.12.004 33575466 PMC7851491

[36] Zhang H , Li Y , Guo S , Wang Y , Wang H , Lu D , et al. Effect of ERCC2 rs13181 and rs1799793 polymorphisms and environmental factors on the prognosis of patients with lung cancer. Am J Transl Res. 2020;12(10):6941–53. 33194084 PMC7653631

[37] Adel Fahmideh M , Schwartzbaum J , Frumento P , Feychting M . Association between DNA repair gene polymorphisms and risk of glioma: a systematic review and meta‐analysis. Neuro Oncol. 2014;16(6):807–14. 10.1093/neuonc/nou003 24500421 PMC4022225

[38] Qian T , Zhang B , Qian C , He Y , Li Y . Association between common polymorphisms in ERCC gene and glioma risk: a meta‐analysis of 15 studies. Medicine (Baltimore). 2017;96(20):e6832. 10.1097/md.0000000000006832 28514298 PMC5440135

[39] Huang LM , Shi X , Yan DF , Zheng M , Deng YJ , Zeng WC , et al. Association between ERCC2 polymorphisms and glioma risk: a meta‐analysis. Asian Pac J Cancer Prev. 2014;15(11):4417–22. 10.7314/apjcp.2014.15.11.4417 24969862

[40] Geng P , Ou J , Li J , Liao Y , Wang N , Xie G , et al. A comprehensive analysis of influence ERCC polymorphisms confer on the development of brain tumors. Mol Neurobiol. 2016;53(4):2705–14. 10.1007/s12035-015-9371-3 26264164

