# Supplementary material for: Genetic variants in XPD gene and glioma susceptibility in Chinese children: A multicenter case–control study
Source: Cancer Innov. 2022 Jun 30;1(1):70–9. doi: 10.1002/cai2.6 (PMC10686151; doi:10.1002/cai2.6)
Supplement: Supplementary file 1 — Supporting information. [file CAI2-1-70-s001.docx]

| **Table S1**. Frequency distribution of selected variables in glioma patients and cancer-free controls in combined subjects | | | | | |
| --- | --- | --- | --- | --- | --- |
| Variables | Cases (N=314) | | Controls (N=380) | | *P* ^a^ |
|  | No. | % | No. | % |  |
| Age range, month | 1.00-168.00 | | 1.00-168.00 | | 0.461 |
| Mean ± SD | 70.46 ± 48.39 | | 64.19 ± 37.38 | |  |
| <60 | 135 | 42.99 | 174 | 45.79 |  |
| ≥60 | 179 | 57.01 | 206 | 54.21 |  |
| Sex |  |  |  |  | 0.379 |
| Female | 146 | 46.50 | 164 | 43.16 |  |
| Male | 168 | 53.50 | 216 | 56.84 |  |
| Subtypes | | | | | |
| Astrocytic tumors | 214 | 68.15 | / | / |  |
| Ependymoma | 61 | 19.43 | / | / |  |
| Neuronal and mixed neuronal-glial tumours | 25 | 7.96 | / | / |  |
| Embryonal tumors | 12 | 3.82 | / | / |  |
| NA | 2 | 0.64 | / | / |  |
| WHO stages | | | | | |
| I | 151 | 48.09 | / | / |  |
| II | 73 | 23.23 | / | / |  |
| III | 36 | 11.46 | / | / |  |
| IV | 53 | 16.88 | / | / |  |
| NA | 1 | 0.32 | / | / |  |
| SD, standard deviation; NA, not available.  ^a^ Two-sided χ^2^ test for distributions between glioma patients and cancer-free controls. | | | | | |
